# Fluoridated Apatite Coating on Human Dentin via Laser-Assisted Pseudo-Biomineralization with the Aid of a Light-Absorbing Molecule

**DOI:** 10.3390/ijms232415981

**Published:** 2022-12-15

**Authors:** Ayako Oyane, Ikuko Sakamaki, Maki Nakamura, Kenji Koga, Kanako Shitomi, Saori Tanaka, Hirofumi Miyaji

**Affiliations:** 1 Nanomaterials Research Institute, National Institute of Advanced Industrial Science and Technology (AIST), Central 5, 1-1-1 Higashi, Tsukuba 305-8565, Japan; 2 Division of Periodontology and Endodontology, Department of Oral Rehabilitation, School of Dentistry, Health Sciences University of Hokkaido, 1757 Kanazawa, Tobetsu-cho, Ishikari-gun 061-0293, Japan; 3 Division of General Dentistry Center for Dental Clinics, Hokkaido University Hospital, N14W5, Kita-ku, Sapporo 060-8648, Japan; 4 Department of Periodontology and Endodontology, Faculty of Dental Medicine, Hokkaido University, N13W7, Kita-ku, Sapporo 060-8586, Japan

**Keywords:** apatite, fluoride, laser, biomineralization, coating, dentin, biomimetic process

## Abstract

A simple, area-specific coating technique for fluoridated apatite (FAp) on teeth would be useful in dental applications. Recently, we achieved area-specific FAp coating on a human dentin substrate within 30 min by a laser-assisted biomimetic (LAB) process; pulsed Nd:YAG laser irradiation in a fluoride-containing supersaturated calcium phosphate solution (FCP solution). The LAB-processed, FAp-coated dentin substrate exhibited antibacterial activity against a major oral bacterium, *Streptococcus mutans*. In the present study, we refined the LAB process with a combination of a dental diode laser and a clinically approved light-absorbing molecule, indocyanine green (ICG). A micron-thick FAp layer was successfully formed on the dentin surface within only 3 min by the refined LAB process, i.e., dental diode laser irradiation in the FCP solution following ICG treatment. The ICG layer precoated on the dentin substrate played a crucial role in inducing rapid pseudo-biomineralization (FAp layer formation) on the dentin surface by absorbing laser light at the solid-liquid interface. In the refined LAB process, the precoated ICG layer was eliminated and replaced with the newly formed FAp layer composed of vertically oriented pillar-like nanocrystals. Cross-sectional ultrastructural analysis revealed a smooth interface between the FAp layer and the dentin substrate. The refined LAB process has potential as a tool for the tooth surface functionalization and hence, is worth further process refinement and in vitro and in vivo studies.

## 1. Introduction

The major inorganic component (biomineral) of human teeth and bones is apatite, a type of calcium phosphate (CaP) compound. Biological apatite is synthesized via CaP precipitation, maturation, and growth in body fluid that is supersaturated with respect to CaPs (a reaction known as biomineralization). Pseudo-biomineralization can be induced in vitro by using acellular supersaturated CaP solutions (e.g., simulated body fluids [1]) as a reaction medium. Such a pseudo-biomineralization process in a supersaturated CaP solution (called a biomimetic process) is useful as a mild, liquid-phase apatite coating technique on materials [2,3,4]. Using biomimetic processes, various materials, including low-melting-point polymers, can be coated with a micron-thick layer of low-crystalline apatite. The resulting apatite layers exhibit good biocompatibility with living soft and hard tissues. Furthermore, biomimetic processes offer biofunctionalized apatite coatings loaded with a variety of biofunctional substances: trace elements, proteins, nucleic acids, vitamins, etc. [3,4]. This is a noteworthy advantage of biomimetic processes over other practical apatite coating techniques, such as plasma spraying, sputtering, and pulsed laser deposition. Biomimetic processes however have low practical utility because they generally require complex and time-consuming procedures (taking hours or days).

Recently, we developed a laser-assisted biomimetic (LAB) process [5] as a simple, area-specific CaP coating technique inspired by a liquid-phase laser irradiation process [6]. In the LAB process, a pulsed Nd:YAG laser is used to irradiate a substrate that is immersed in a supersaturated CaP solution. That process enables rapid CaP coating (irradiation time ≤ 30 min) on target surfaces of some polymeric, ceramic, and metallic materials via pseudo-biomineralization induced by the effects of laser surface modification and heating [5,7,8]. LAB-processed, apatite-coated polymer substrates show improved cell compatibility compared to the untreated substrates [7]. More recently, we applied the LAB process (irradiation time = 30 min) to a human dentin substrate and succeeded in functionalizing its surface with micron-thick layers of apatite [9] and fluoridated apatite (FAp) [10]. The layers formed on the substrate were both comprised of *c*-axis-oriented pillar-like nanocrystals and were integrated seamlessly with the dentin. The LAB-processed, FAp-coated dentin substrate suppressed the proliferation of a major oral bacterium *Streptococcus mutans* [10], most likely due to the antimicrobial effect of fluoride ions [11] released from the FAp layer. These results show the potential of the LAB process as a tool for tooth surface functionalization.

Other FAp or apatite coating techniques on teeth include precipitation processes with [12,13] and without [14,15,16,17,18,19] lasers, powder jet deposition [20], pulsed laser deposition [21], film-fusion [22], and laser-induced forward transfer [23]. However, each of these techniques has its advantages and drawbacks, and it is still difficult to find a practical technique that meets all the requirements for chair-side FAp coating on teeth: safety, area-specific coating capability, simplicity, easy handling, and swiftness.

The aim of the present study was to refine the LAB process with the use of a dental diode laser for rapid FAp coating on the dentin substrate. To move closer to clinical applications, we employed a clinically available near-infrared (NIR) dental diode laser (λ = 808 ± 10 nm) with continuous wave (cw). NIR dental diode lasers are widely used for clinical treatment (e.g., cutting) of oral soft tissues. However, NIR laser light is hardly absorbed by human dentin substrates, although laser absorption by the substrate is crucial in the LAB process for inducing pseudo-biomineralization on the surface [5,8]. We hypothesized that application of an NIR-absorbing molecule, indocyanine green (ICG) onto the dentin substrate should enable rapid pseudo-biomineralization on the surface under NIR laser irradiation. ICG is an amphiphilic molecule that has long been used as an NIR fluorescence dye for medical diagnostics because of its unique features; it efficiently absorbs NIR light and is metabolized in the body with minimal toxicity [24]. ICG has also attracted much attention as an NIR-absorbing molecule for laser-mediated photodynamic therapy [25]. Here, we propose a refined LAB process with a combination of a dental diode laser and a clinically approved light-absorbing molecule ICG.

## 2. Results

### 2.1. Surface Analyses of the ICG-Treated Substrate

**Figure 1:**
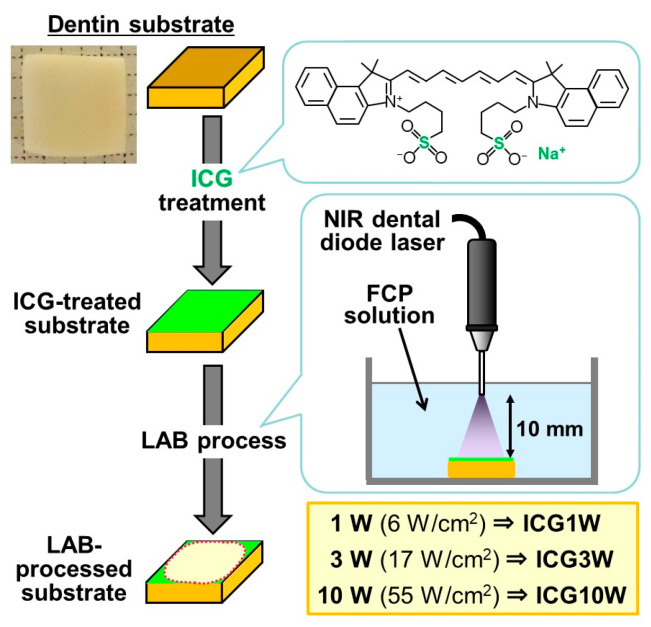
Digital camera image of an untreated human dentin substrate (top-left, grid = 1 mm) and schematic of the refined LAB process: NIR dental diode laser irradiation at various powers for 3 min of the dentin substrate in an FCP solution following ICG treatment.

Figure 1 shows a schematic of the refined LAB process with the aid of ICG. First, a human dentin substrate was subjected to ICG treatment in which an ICG solution was applied onto the dentin surface and air-dried. Second, ICG-treated and untreated (as a control) substrates were immersed in a fluoride-containing supersaturated CaP solution (FCP solution) and irradiated on the surface with an NIR dental diode laser under different conditions for 3 min (the LAB process). The structures and compositions of the ICG-treated and LAB-processed dentin surfaces were examined by surface and cross-sectional analyses.

**Figure 2:**
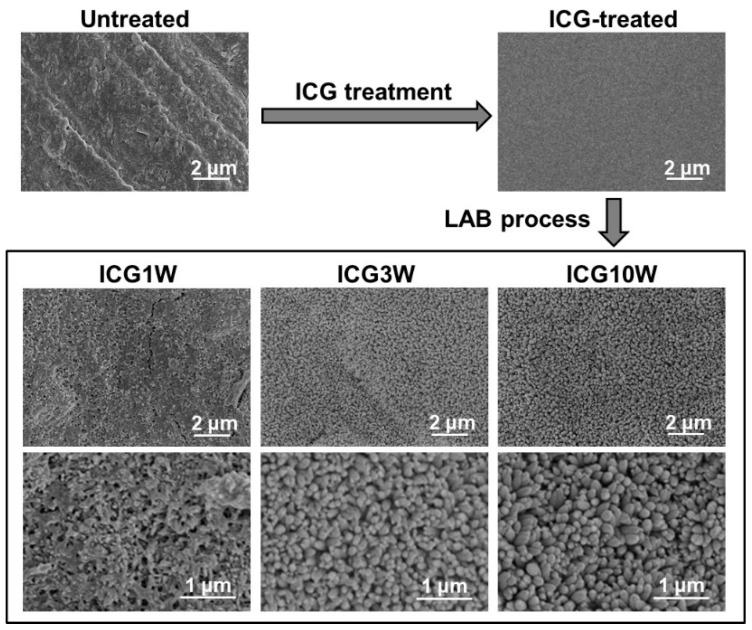
SEM images of the surfaces of the untreated substrate (top-left), ICG-treated substrate (top-right), and LAB-processed substrates: ICG1W (left), ICG3W (middle), and ICG10W (right), with lower (middle row) and higher (bottom row) magnification.

**Figure 3:**
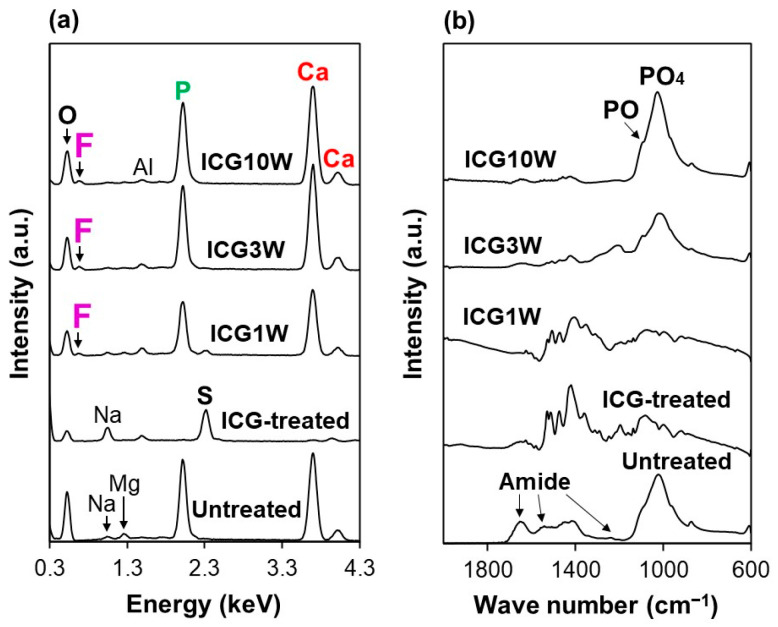
(**a**) SEM-EDX and (**b**) FT-IR spectra of the surfaces of the untreated substrate, ICG-treated substrate, and LAB-processed substrates: ICG1W, ICG3W, and ICG10W. The small Al peak in (**a**) is derived from the sample holder.

The ICG-treated substrate had a dense and smooth ICG layer on its surface according to the results of scanning electron microscopy (SEM), energy-dispersive X-ray (EDX) analysis, and Fourier transform infrared spectroscopy (FT-IR) in conjunction with an attenuated total reflection (ATR) accessory with a monolithic diamond crystal. As apparent in the top SEM images in Figure 2, the untreated dentin substrate with a micron-rough surface became flattened after the ICG treatment. According to the SEM-EDX analysis (Figure 3a), component elements of dentinal apatite (Ca, P, and O) were clearly detected on the untreated substrate. Among these elements, Ca and P disappeared while component elements specific to ICG (S and Na) became distinct after the ICG treatment. In the FT-IR spectrum of the untreated substrate (Figure 3b), peaks ascribed to amide and phosphate groups (from dentinal collagen and apatite, respectively) were detected. After the ICG treatment, new peaks ascribed to ICG became dominant rather than the peaks derived from dentin. These analytical results indicated that the dentin substrate was precoated with a dense and smooth ICG layer by the ICG treatment.

### 2.2. Surface Analyses of the LAB-Processed Substrate

In the LAB process, the ICG-precoated substrate underwent different surface morphological changes depending on the laser power. The substrates subjected to the ICG treatment and subsequent LAB process for 3 min with an output power of 1 W (nominal power density at the dentin surface = 6 W/cm^2^), 3 W (17 W/cm^2^), and 10 W (55 W/cm^2^) are referred to as ICG1W, ICG3W, and ICG10W, respectively (see Figure 1). As shown in Figure 2, the smooth surface of the ICG-precoated substrate became roughened at a submicron scale after the LAB process at 1, 3, and 10 W. The magnified images (bottom row) revealed that ICG3W and ICG10W had densely packed nanoparticles on their surfaces, whereas ICG1W had a submicron porous structure on its surface. The nanoparticles on the ICG10W surface were apparently larger than those on the ICG3W surface.

The nanoparticles found on the ICG3W and ICG10W surfaces were composed of F-incorporated CaP according to the SEM-EDX and FT-IR results. For ICG3W and ICG10W, peaks ascribed to the precoated ICG layer were barely detected either by SEM-EDX (Figure 3a) or FT-IR (Figure 3b). The SEM-EDX and FT-IR spectra of ICG3W and ICG10W were both similar to those of the untreated substrate except for the following two differences. First, fluoride was detected on the ICG3W and ICG10W surfaces by SEM-EDX, although it was not detected on the untreated dentin surface (Figure 3a). Second, ICG3W and ICG10W showed increased intensity of phosphate peaks (from apatite) relative to the amide peaks (from dentinal collagen) compared to the untreated dentin surface (Figure 3b). The observed changes in the LAB process at 3 and 10 W might have been caused by the thinning of the precoated ICG layer and precipitation of F-incorporated CaP on the dentin surface, as described in Section 2.3.

The LAB process at lower power (1 W) caused thinning of the precoated ICG layer without inducing significant CaP precipitation on the substrate (ICG1W). In the SEM-EDX spectra (Figure 3a), Ca and P peaks appeared while S and Na peaks ascribed to ICG diminished after the LAB process at 1 W. In contrast, no remarkable change was observed in their FT-IR spectra (Figure 3b). Considering the higher surface sensitivity of FT-IR (with ATR) than of SEM-EDX, the precoated ICG layer remained on the ICG1W surface although it became thinner after the LAB process at 1 W. The residual ICG layer on the ICG1W surface contained no or only a small quantity of CaP, because phosphate peaks were not apparent in its FT-IR spectrum. These results suggest that the morphological change observed on the ICG1W surface (Figure 2) was mainly due to laser etching of the precoated ICG layer rather than CaP precipitation.

### 2.3. Cross-Sectional Analyses of the LAB-Processed Substrate

**Figure 4:**
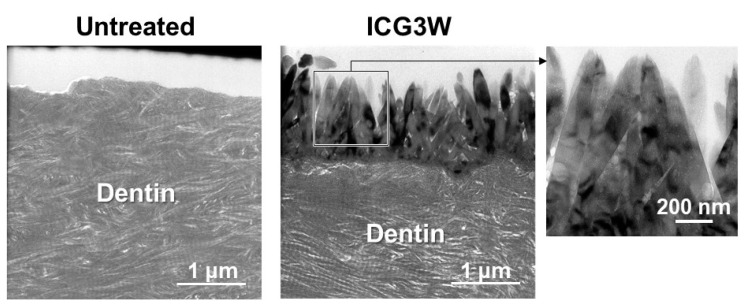
Cross-sectional TEM images of the surfaces of the untreated substrate (left) and LAB-processed substrate: ICG3W (middle, right), with lower (left, middle) and higher (right) magnification.

The F-incorporated CaP particles found on the ICG3W surface had a pillar-like morphology, were oriented vertically to the dentin surface, and constituted a micron-thick layer that integrated seamlessly with the underlying dentin. Cross-sectional transmission electron microscopy (TEM) analysis was performed on the untreated substrate and the LAB-processed substrate: ICG3W. Cross-sectional samples were prepared from the substrates by focused ion beam (FIB) fabrication. The results revealed that the bottom dentinal regions were apparently similar between the untreated and LAB-processed substrates; both showed a periodic (~67 nm) D-band structure [26,27] characteristic of type I collagen fibrils, along with fine needle-like structures characteristic of dentinal apatite (Figure 4). In contrast, the surfaces of the two substrates showed large differences; the untreated substrate had a relatively smooth surface, whereas ICG3W had a submicron-rough surface owing to the presence of the pillar-like particles with minor and major axes of 0.1–0.2 μm and 1.0–1.4 μm, respectively. These pillar-like particles were packed densely and oriented vertically to the dentin surface; therefore, their minor axes corresponded to the particle diameters in the top-view SEM image (bottom middle image in Figure 2) of the ICG3W surface. Note that the assembled pillar-like particles constituted a micron-thick layer that covered the dentin surface seamlessly.

**Figure 5:**
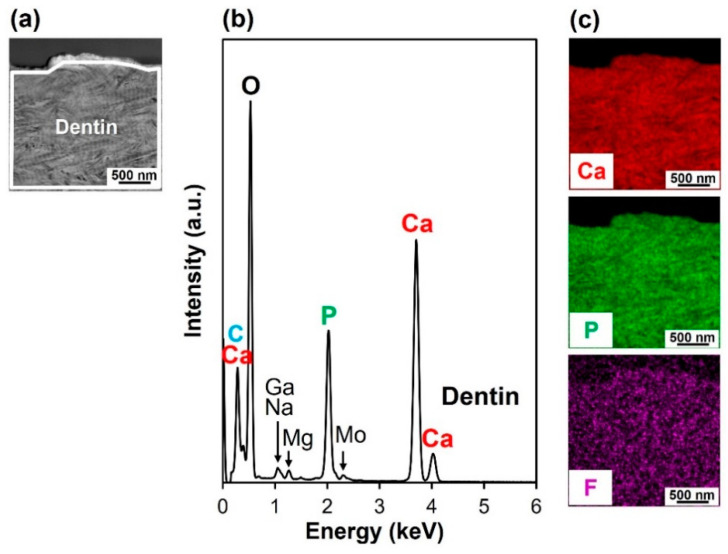
(**a**) Cross-sectional HAADF-STEM image of the surface of the untreated substrate, (**b**) STEM-EDX spectrum obtained from the boxed region in (**a**), and (**c**) STEM-EDX elemental (Ca, P, F) maps of (**a**).

**Figure 6:**
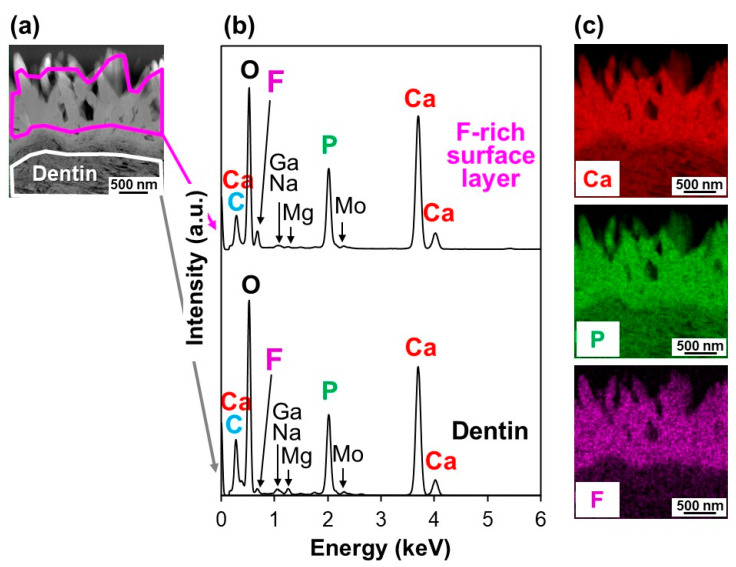
(**a**) Cross-sectional HAADF-STEM image of the surface of the LAB-processed substrate ICG3W, (**b**) STEM-EDX spectra obtained from the boxed regions in (**a**), and (**c**) STEM-EDX elemental (Ca, P, F) maps of (**a**).

The micron-thick layer on the ICG3W surface was composed of F-incorporated CaP according to the cross-sectional EDX analysis using a high-angle annular dark field (HAADF) scanning TEM (STEM) system, which reconfirmed the SEM-EDX and FT-IR results (Figure 3). Figure 5b and Figure 6b show cross-sectional STEM-EDX spectra of the surfaces of the untreated substrate and LAB-processed substrate (ICG3W), respectively. Each spectrum was obtained respectively from the boxed region in the corresponding HAADF-STEM images in Figure 5a and Figure 6a. The dentinal region in the two substrates displayed small peaks due to Ga, Na, Mg, and Mo, in addition to the strong peaks due to O, P, and Ca. Heavy metal elements Ga and Mo are contaminants derived from the FIB process and TEM grid, respectively, and other elements are from the dentin. All the spectra in Figure 5b and Figure 6b were basically similar to each other except for the F peak intensity; the F peak intensity of the surface CaP layer on ICG3W was higher than that of the dentinal regions in the untreated substrate and ICG3W. In ICG3W, the F/P atomic ratio (0.25 ± 0.00) of the surface CaP layer was higher than that (0.04 ± 0.02) of the underlying dentinal region. The observed regional difference in the F content is visualized clearly in the STEM-EDX elemental maps shown in Figure 6c; the surface CaP layer was rich in F compared to the underlying dentinal region. These STEM-EDX results agree well with the SEM-EDX results (Figure 3a), reconfirming the incorporation of F within the CaP layer on ICG3W. Note that the component element (S) specific to ICG was hardly detected on the ICG3W surface, either in the surface layer or in the underlying dentinal region (Figure 6b). This result indicates that the ICG layer precoated on the substrate was eliminated and replaced with the F-incorporated CaP layer in the LAB process at 3 W.

### 2.4. Crystalline Phase Analyses of the Layer on the LAB-Processed Substrate

**Figure 7:**
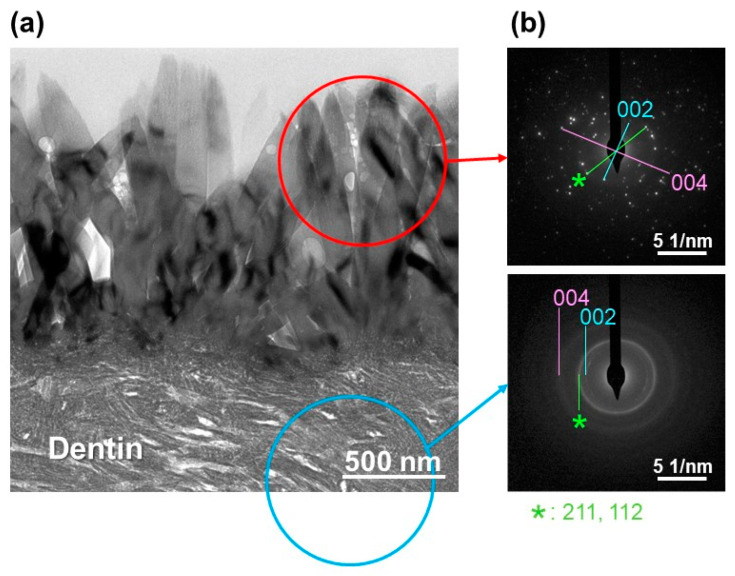
(**a**) Cross-sectional TEM image of the surface of the LAB-processed substrate ICG3W, and (**b**) SAED patterns obtained from the circled regions in (**a**). * in (**b**) corresponds to apatite 211 and 112 diffraction spots or rings.

**Figure 8:**
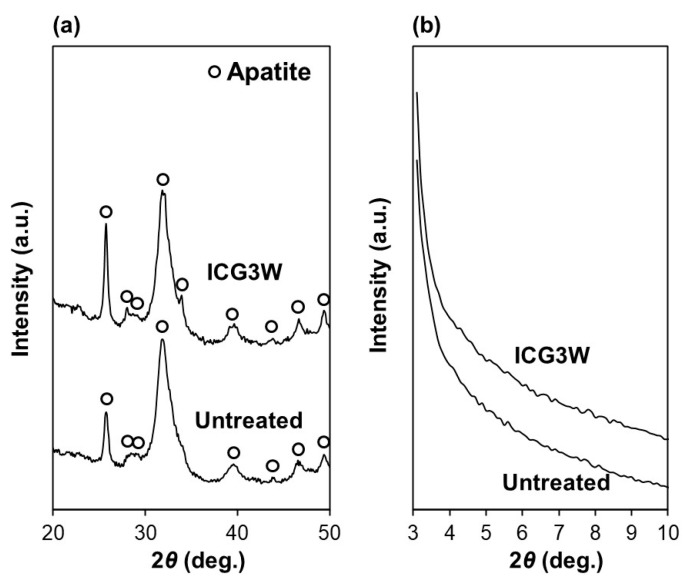
XRD patterns with (**a**) wide and (**b**) small angle regions of the surfaces of the untreated substrate and LAB-processed substrate ICG3W. The XRD patterns of both untreated and LAB-processed substrates were obtained from the same substrate to prevent variations due to individual differences among dentin substrates.

The F-incorporated CaP layer on the ICG3W surface was identified as crystalline FAp according to the selected area electron diffraction (SAED) and X-ray diffraction (XRD) analyses. Figure 7a,b show cross-sectional TEM image of the ICG3W surface and SAED patterns obtained from the circled regions in Figure 7a, respectively. Both the F-incorporated CaP layer on the ICG3W surface and the underlying dentinal region gave SAED patterns ascribed to crystalline apatite. The surface FAp layer gave a spotty SAED pattern indicating a single crystalline pillar-like structure, while the dentinal apatite gave a concentric ring pattern denoting a polycrystalline structure. The XRD analysis was performed for the same dentin substrate to prevent variation due to individual differences among substrates. As shown in Figure 8a, all the diffraction peaks from the untreated substrate and ICG3W were indexed to apatite. Octacalcium phosphate (OCP; main peak position at 4.7°) was under the detection limit for both substrates (Figure 8b). Some of the diffraction peaks of apatite (at 26, 28, and 34°) shown in Figure 8a were sharper for ICG3W than for the untreated substrate. This difference can be attributed to the presence of well-developed FAp crystals compared to the dentinal apatite on the ICG3W surface, as seen in the TEM results (Figure 7). After the LAB process, the apatite 002 diffraction peak at 26° notably increased compared to other apatite peaks (Figure 8a), most likely due to the c-axis orientation of the FAp crystals along the surface normal as reported in our previous report [10]. Taken together, the dentin substrate was coated on its surface with the FAp layer by the LAB process at 3 W.

### 2.5. Comparative Study

**Figure 9:**
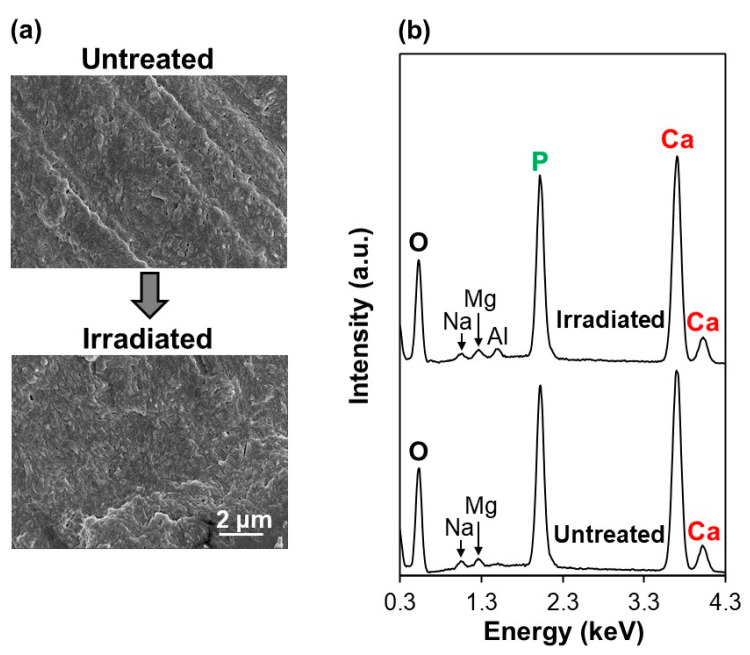
(**a**) SEM images and (**b**) SEM-EDX spectra of the surfaces of the untreated substrate and that after laser irradiation (3 W) for 3 min in the FCP solution without prior ICG treatment. The small Al peak in (**b**) is derived from the sample holder.

The ICG layer precoated on the dentin substrate played a crucial role in the formation of the FAp layer on the dentin surface in the present LAB process. As revealed by the SEM-EDX analysis, without prior ICG treatment, the dentin substrate showed no apparent changes either in its surface morphology (Figure 9a) or chemical composition (Figure 9b) under laser irradiation at 3 W for 3 min. There was no sign of CaP precipitation on the dentin surface without ICG even after the same irradiation process used for ICG3W.

## 3. Discussion

### 3.1. Putative Mechanism of FAp Formation in the Present LAB Process

The human dentin substrate was successfully coated with a micron-thick FAp layer by the present LAB process using the NIR dental diode laser in combination with the NIR-absorbing molecule ICG (Figure 2, Figure 3, Figure 4, Figure 6, Figure 7 and Figure 8). While the mechanism of FAp formation in the present LAB process is still unclear, we consider that the ICG layer precoated on the dentin substrate absorbed laser light energy, thereby inducing rapid pseudo-biomineralization (FAp layer formation) as described below.

In the LAB process at 3 W, the precoated ICG layer, initially with a smooth surface (top-right image in Figure 2), disappeared almost entirely from the dentin surface (Figure 3 and Figure 6). This might be due to photothermal degradation and ablation of ICG under laser irradiation. While the ICG layer is thinned under laser irradiation, thermal energy generated in the layer should be diffused to heat the neighboring FCP solution. In the heated FCP solution, chemical species (ions and CaP nanoclusters [28,29]) gain greater kinetic energy, while the degree of supersaturation with respect to FAp increases further owing to the reduced solubility of FAp [30], both of which have an accelerating effect on CaP precipitation. The precipitation of CaP should be induced on dentinal collagen as well as dentinal apatite crystals (as seed crystals), which were exposed and activated on the substrate surface following the elimination of the precoated ICG layer under laser irradiation. The ICG residues remaining on the substrate surface might also be involved in the initial precipitation step. The precipitated CaP nanoparticles grew into pillar-like crystals under laser irradiation and finally formed a micron-thick FAp layer after the LAB process for 3 min (Figure 2, Figure 3, Figure 4, Figure 6, Figure 7 and Figure 8). The expected composition of the final FAp layer was Ca_10_(PO_4_)_6_(OH)_0.5_F_1.5_ according to the layer’s F/P atomic ratio (0.25, calculated in Section 2.3), assuming that FAp has the above stoichiometric composition without any other ionic substitution. In the final FAp layer, the pillar-like crystals were densely packed, and their longer axes were oriented vertically to the dentin surface (Figure 4 and Figure 7). The observed crystal orientation results from a mechanism known as *crystal growth selection* (or geometric selection of crystals); in crystal growth from a large number of surface nucleation sites, crystals growing vertically to the surface can reach larger sizes since others stop growing due to crystal impingement [31]. Similar crystal orientation was observed also in the apatite [9] and FAp [10] layers formed by our previous LAB process without using ICG.

In the LAB process with lower (1 W) and higher power (10 W), the series of reactions described above was decelerated and accelerated, respectively. Thus, the ICG1W surface was comprised mainly of the residual ICG layer, whereas the ICG10W surface was comprised of larger FAp-like nanoparticles (Figure 2 and Figure 3).

Without prior ICG treatment, no noticeable changes were observed on the dentin surface due to laser irradiation at 3 W in the FCP solution for 3 min (the same irradiation condition used for ICG3W) (Figure 9). This indicates that, despite the presence of dentinal apatite crystals on the dentin substrate, they hardly grew in the FCP solution in the tested immersion period (3 min) even under laser irradiation. This is because of the low laser light absorption by the bare dentin substrate (without ICG) and slow FAp growth rate in the FCP solution without any stimulation. Thus, the presence of ICG on the dentin surface was a prerequisite in the present LAB process for inducing rapid pseudo-biomineralization (FAp layer formation) on the dentin surface, although the precoated ICG layer was eliminated in the process.

### 3.2. Comparison with Previous Biomimetic Processes

The present LAB process using the NIR dental diode laser following ICG treatment enabled rapid FAp coating on the human dentin substrate. In conventional biomimetic processes without laser irradiation, a substrate is surface-modified with nucleating agents and/or CaP seeds, and then immersed in a supersaturated CaP solution for hours or days to grow a micron-thick apatite layer on the surface [4]. For example, a CaP-precoated substrate forms a 3 µm-thick apatite layer on its surface after immersion in a supersaturated CaP solution (without fluoride ions) for 17 h [32]. In our previous LAB process without using ICG, FAp nanocrystals a few tens of nanometers in size formed on the dentin surface within 5 min in the FCP solution, and then grew into a micron-thick (2−2.5 μm) FAp layer within 30 min under laser irradiation (30 Hz, 6 W/cm^2^) [10]. In the present LAB process, even faster FAp formation was achieved; a micron-thick (1−1.5 μm) FAp layer was formed on the ICG-treated dentin surface within only 3 min under laser irradiation (Figure 4). The use of a higher power laser (cw, 17 W/cm^2^) in combination with the light-absorbing molecule ICG might accelerate the precipitation and growth of FAp on the dentin surface. The present LAB process would be more practical than the previous one [10] in terms of irradiation time, although a prior ICG treatment step is needed.

### 3.3. Limitations and Future Perspectives

There are two major limitations in this study. First, mechanism of FAp formation in the present LAB process is yet to be fully clarified. For instance, surface structural changes with shorter irradiation time (than 3 min) and the role of dentinal apatite crystals remain unclear. More detailed studies including a time-course study are needed for the elucidation of the reaction mechanism and further process refinement.

Second, the safety and efficacy of the present LAB process for tooth surface functionalization are still unclear. The dental diode laser and ICG used in the present LAB process are a clinically approved medical device and injectable fluorescence dye, respectively. This laser has been used for cutting, hemostasis (elimination of bleeding), coagulation, and ablation of oral soft tissues such as gingiva, and ICG has been used for clinical imaging. However, neither of them is yet approved for this proposed use, i.e., FAp coating on teeth. Thus, the present LAB process may have adverse effects on teeth and/or periodontal tissues, which should be addressed in future research. Regarding efficacy, we expect that an LAB-processed FAp-coated tooth surface will exhibit not only antibacterial activity but also improved acid resistance. It is known that fluoride ions exhibit antimicrobial effects [11] along with tooth-strengthening effects via ionic substitution of the hydroxide site in dentinal hydroxyapatite [33]. In fact, the FAp layer produced by the previous LAB process without using ICG showed antibacterial activity against *Streptococcus mutans* [8,10] and superior acid resistance to sintered hydroxyapatite [8]. This FAp layer was similar to the FAp layer produced by the present LAB process; both layers were a few micrometers in thickness, integrated smoothly with the dentin substrate, and comprised of well-developed, vertically oriented pillar-like FAp crystals. It is thus highly anticipated that the FAp layer produced by the present LAB would also exhibit antibacterial activity and acid resistance, like the FAp layer produced by the previous LAB process. Further in vitro and in vivo studies are needed to verify this hypothesis. Safety assessment, and mechanical and biochemical property evaluation are also required to assess the potential of the present LAB process as a tool for tooth surface functionalization.

## 4. Materials and Methods

### 4.1. Preparation of Human Dentin Substrates

Experiments using human dentin substrates were conducted under conditions approved by the ethical review boards of Hokkaido University Hospital, Health Sciences University of Hokkaido, and National Institute of Advanced Industrial Science and Technology (AIST). After obtaining informed consent, we received tooth roots of vital third molars which were extracted in usual dental treatments from male and female patients older than 20 years at the dental department of Hokkaido University Hospital. Tooth roots severely contaminated by periodontal disease or root caries were excluded from the study. From donated third molar roots, 1 mm-thick dentin substrates with dimensions of 3–5 mm × 5 mm were prepared using a diamond disk (Horico diamond disk 87xFSI, HORICO DENTAL Hopf, Ringleb & Co. GmbH & Cie., Berlin, Germany). The dentin substrates were polished on their surfaces with #600 and #2000 SiC polishing papers, ultrasonically washed with pure water, and stored in a freezer (−20 °C) before use. The prepared dentin substrate (placed on 1 mm graph paper) was captured using a digital camera (TG-5; Olympus Corporation, Tokyo, Japan), and shown in the top left image in Figure 1.

### 4.2. Preparation of a Fluoride-Free Supersaturated CaP Solution (Mother Solution)

All the experiments described in Section 4.2, Section 4.3 and Section 4.4 were conducted in an air-conditioned laboratory at approximately 25 °C. As a mother solution for the FCP solution, we prepared a fluoride-free supersaturated CaP solution using special grade chemicals (all from NACALAI TESQUE, Inc., Kyoto, Japan) as described previously [7,8,9,10]. Briefly, the CaP solution was prepared by dissolving NaCl (142 mM), K_2_HPO_4_·3H_2_O (1.50 mM), 1 M HCl (40 mM), and CaCl_2_ (3.75 mM) in ultrapure water and adjusting the final pH to 7.40 at 25 °C with tris(hydroxymethyl)aminomethane (50 mM) and a 1 M HCl solution (amount required for the pH adjustment). The CaP solution was a colorless and transparent solution and stored in a refrigerator (4 °C) up to 4 weeks before use.

### 4.3. Treatment with ICG

The dentin substrates (prepared in Section 4.1) were treated with ICG (Diagnogreen for injection 25 mg, Daiichi Sankyo Company, Ltd., Tokyo, Japan) prior to the LAB process (Section 4.4). According to the manufacturer’s instruction, 25 mg of ICG in a vial was dissolved in 0.5 mL of water for injection to prepare an ICG solution. The ICG solution was applied to the surface (one side only, 0.2 μL/mm^2^) of each dentin substrate. The substrate was placed in a light-shielding container while drying in the air for 20 min. After drying, the ICG-treated substrate was subjected immediately to the LAB process (Section 4.4).

### 4.4. LAB Process for FAp Coating

Just before irradiation, the FCP solution (same solution as “F1000” in Reference [8]) was prepared by dissolving NaF (1.0 mM) (NACALAI TESQUE, Inc.) in the fluoride-free supersaturated CaP solution (prepared in Section 4.2), followed by filtration with a 0.22-μm pore-size filter. The ICG-treated substrate (prepared in Section 4.3) or the untreated substrate (prepared in Section 4.1) for comparative study was placed in a poly(tetrafluoroethylene) sample holder in 10 mL of the FCP solution. The substrate surface was irradiated for 3 min with NIR laser light (cw, λ = 808 ± 10 nm) using a dental diode laser (S LASER, GC Corporation, Tokyo, Japan) through an optical fiber with a core diameter of 300 μm (Figure 1). The distance between the substrate surface and the fiber edge was adjusted to 10 mm. In this irradiation system, the nominal beam diameter at the substrate surface was approximately 5 mm. The laser output power was set at 1 W (nominal power density at the dentin surface = 6 W/cm^2^), 3 W (17 W/cm^2^), and 10 W (55 W/cm^2^). After laser irradiation for 3 min, the substrate was removed from the FCP solution, washed gently with ultrapure water, and dried in the air before analyses.

### 4.5. Surface Analyses

The surfaces of the substrates were examined by SEM (S-4800, Hitachi High-Tech Corporation, Tokyo, Japan), SEM-EDX analysis (AZtec-One, Oxford Instruments plc, Abingdon, England) with a tabletop SEM (TM4000Plus II, Hitachi High-Tech Corporation), FT-IR (FT/IR-4700, JASCO Corporation, Hachioji, Japan) in conjunction with an ATR accessory with a monolithic diamond crystal, and XRD (M18X, MacScience, Yokohama, Japan) analysis with CuKα radiation. For the LAB-processed substrates, laser-irradiated regions on their surfaces were used for the SEM, SEM-EDX, and FT-IR analyses. In the SEM-EDX analysis, the substrates were fixed on an aluminum holder and analyzed without coating. Before the SEM analysis, the substrates were sputter-coated on their surfaces with electroconductive gold. In the XRD analysis, the same substrate (untreated substrate and that after the LAB process) was used to prevent variation due to individual differences among dentin substrates.

### 4.6. Cross-Sectional Analyses

Cross-sectional samples for TEM analysis were prepared from selected substrates (untreated substrate and ICG3W) by the FIB process with a gallium ion source (FB-2100, Hitachi High-Tech Corporation). Prior to the FIB process, oily ink was applied to the substrate to protect its surface, and tungsten was deposited on the substrate surface using W(CO)_6_ gas. From the surface portion of the substrate, a cross-sectional sample was picked-up, fixed on a molybdenum FIB lift-out grid, and then thinned to approximately 100 nm. The cross-sectional samples were analyzed using an analytical TEM system (Tecnai Osiris, FEI, Hillsboro, OR, USA) operating at 200 kV in conjunction with an EDX spectrometer (Super-X system) and a HAADF-STEM system with an electron probe smaller than 1 nm in diameter. In the compositional analysis by STEM-EDX, the average and standard deviation values were calculated using data obtained from three different regions.

## 5. Conclusions

A micron-thick FAp layer was formed on the human dentin substrate by the present LAB process, i.e., NIR dental diode laser irradiation (17 W/cm^2^, 3 min) in the FCP solution following treatment with the NIR-absorbing molecule ICG. The ICG layer precoated on the dentin substrate absorbed laser light energy in the LAB process and played a crucial role in inducing rapid pseudo-biomineralization (FAp layer formation) on the dentin surface. In the LAB process, the precoated ICG layer was eliminated and replaced with the newly formed FAp layer composed of vertically oriented pillar-like nanocrystals. Cross-sectional ultrastructural analysis revealed a smooth interface between the FAp layer and the dentin substrate. The present LAB process is worth further process refinement and in vitro and in vivo studies to assess its potential as a tool for tooth surface functionalization.

## Data Availability

Not applicable.
